# Absolute Radiometric Calibration of TESS-W and SQM Night Sky Brightness Sensors

**DOI:** 10.3390/s19061336

**Published:** 2019-03-17

**Authors:** Salvador Bará, Carlos E. Tapia, Jaime Zamorano

**Affiliations:** 1 Departmento Física Aplicada, Universidade de Santiago de Compostela, 15782 Santiago de Compostela, Galicia, Spain; 2 Departamento Física de la Tierra y Astrofísica. Instituto de Física de Partículas y del COSMOS (IPARCOS), Universidad Complutense, 28040 Madrid, Spain; ceta@ucm.es (C.E.T.); jzamorano@fis.ucm.es (J.Z.)

**Keywords:** night sky brightness, radiance, astronomical magnitudes, light pollution

## Abstract

We develop a general optical model and describe the absolute radiometric calibration of the readings provided by two widely-used night sky brightness sensors based on irradiance-to-frequency conversion. The calibration involves the precise determination of the overall spectral sensitivity of the devices and also the constant *G* relating the output frequency of the light-to-frequency converter chip to the actual band-weighted and field-of-view averaged spectral radiance incident on the detector (brightness). From these parameters, we show how to define a rigorous astronomical absolute photometric system in which the sensor measurements can be reported in units of magnitudes per square arcsecond with precise physical meaning.

## 1. Introduction

The brightness of the night sky is significantly higher than what would be expected for a natural night in many regions of the world, due to the atmospheric scattering of artificial light [1,2]. The measurement and modelling of this phenomenon, mostly caused by the emissions of outdoor lighting systems, is, nowadays, the subject of an intense research effort. One of the aims of that work is to acquire the observational data needed to validate (or otherwise reject) the existing models of artificial light propagation throughout the atmosphere (see [3] for a review). A complementary goal is establishing a comprehensive reference dataset with enough statistical power, to be used as a baseline for detecting the changes that the artificial disruption of the night sky darkness may experience in forthcoming years. This is a relevant issue both for basic science and for applications in different fields of knowledge including, among others, the protection of existing and potential astronomical observatory sites [4,5], biodiversity preservation and ecosystem services management [6,7,8], urban emissions monitoring [9,10], energy economics [11,12], as well as for preserving the night sky as a key asset of the intangible cultural heritage of humanity [13].

The emissions of the artificial light sources can be partially monitored using instruments on Earth orbiting platforms, like the Visible Infrared Imaging Radiometer Suite Day/Night Band (VIIRS-DNB) onboard the Suomi National Polar-orbiting Partnership (Suomi-NPP) satellite [14], the digital single-lens reflex (DSLR) RGB images captured by the Crew Earth Observation facility of the International Space Station [15,16], or the new generations of nighttime Earth monitoring satellites [17,18]. However, evaluating the effects of these emissions on the night sky brightness under changing meteorological conditions requires extensive ground-based observations. Several radiance and luminance meters are routinely used for this purpose (for a review, see [19]). Relevant information is also provided by citizen science programs of naked-eye observations of the night sky, like the U.S. National Optical Astronomy Observatory’s Globe at Night [20]. These naked-eye estimations are based on the fact that the number of visible stars decreases monotonically with the increasing artificial brightness of the sky’s background, due to the luminance contrast threshold of the human visual system. Most of the available night sky brightness data, however, are obtained by means of the widely used night sky brightness meters based on irradiance-to-frequency converter chips, like the Sky Quality Meter (SQM) of Unihedron (Grimsby, ON, Canada) [21], or the Telescope Encoder and Sky Sensor (TESS-W) developed by the European Union’s Stars4All project [22]. These devices constitute the backbone of the night sky brightness monitoring networks of several academic institutions [23,24,25,26] and public meteorological agencies [27].

The readings provided by these sensors are usually expressed in magnitudes per square arcsecond (mag/arcsec^2^), a negative logarithmic unit system for filter-weighted radiances commonly used in astronomy and astrophysics. However, the exact definition of the device-specific magnitude scales for these kinds of sensors and their relationship with the actual incident radiance have not been thoroughly addressed in the literature. Whilst this lack of definition does not completely prevent the use of these sensors for detecting qualitative overall trends in the evolution of the anthropogenic night sky brightness, it makes the quantitative comparison of their measurements against the predictions of different atmospheric light propagation models difficult. In order to fill this void, the purpose of this paper is threefold: (i) to develop a formal optoelectronic model for this kind of sensors, (ii) to describe their absolute radiometric calibration procedure, and (iii) to show how to define a rigorous photometric absolute (AB) magnitude system that allows to assign a definite physical meaning to their mag/arcsec^2^ readings in terms of the incident spectral radiance. The sensor modelling and its absolute calibration procedure, as well as the definition of the AB magnitude system, are described in Section 2. In Section 3 we present the particular results obtained for the TESS-W and SQM (model USB enabled data-logging light meter, SQM-LU-DL) devices. Additional remarks are included in Section 4, and overall conclusions drawn in Section 5. Some formal mathematical steps leading to the key modelling equations are described in Appendix A.

## 2. Materials and Methods

### 2.1. Detector Modelling

Irradiance-to-frequency based night sky radiance meters, examples of which are the SQM-LU-DL and TESS-W detectors, are basically composed of (i) an optical block that limits and defines their effective field-of-view, (ii) a monolithic irradiance-to-frequency converter chip whose electrical output is a square wave whose frequency depends linearly on the irradiance incident on the chip surface, (iii) one or several optical filters that, combined with the spectral transmittance of the remaining optical components and the spectral responsivity of the detector, determine the overall spectral sensitivity of the device, and (iv) basic data processing electronics for converting the frequency to the desired radiometric magnitudes.

Both the SQM-LU-DL and the TESS-W use a TSL237 light-to-frequency converter (ams AG, Premstaetten, Austria) [28], that combines a silicon photodiode and a current-to-frequency converter on a single monolithic CMOS integrated circuit, and whose frequency response is linear across several decades of incident irradiance. Their fields of view are limited to a region of the sky with approximately a Gaussian profile and full-width-at-half-maximum (FWHM) of 20° for the SQM-LU-DL and 17° for the TESS-W. The SQM-LU-DL uses a Hoya CM-500 filter for nominally limiting its effective bandpass to 400–650 nm (FWHM 250 nm) [21], whereas the TESS-W is fitted with a dichroic filter that limits it to the 400–740 nm spectral band [22].

Irrespectively of the particular construction details of each device, it can be shown that the output frequency f (Hz) provided by the converter is related to the radiance at the entrance aperture of the detector by the general expression (see Appendix A for details):

(1) $$f=K\;\int_{\lambda =0}^{\infty }\;T\left(\lambda \right)\;\left[\int_{\Omega }P\left(\omega \right)L_{\lambda }\left(\omega \right)d^{2}\omega \right]\;d\lambda +f_{D}$$

where $L_{\lambda }\;\left(\omega \right)$ is the spectral radiance (Wm^−2^sr^−1^nm^−1^) of the incident light field along the direction specified by the angular vector ω=(θ, ϕ), such that $L_{\lambda }\;\left(\omega \right)\;d\lambda$ is the radiance (Wm^−2^sr^−1^) contained within the spectral interval [λ, λ+dλ]. The two-dimensional differential factor $d^{2}\omega$ is the elementary solid angle (sr) around the direction ω (in case of using spherical coordinates with the Z axis along the central ray of the field-of-view $d^{2}\omega =\sin\theta \;d\theta \;d\phi$), and P(ω) is the weighting function describing the field-of-view of the device (units sr^−1^), normalized such that $\int_{\Omega }P\left(\omega \right)\;d^{2}\omega =1$, where Ω stands for the angular half-space subtended by the forward-facing hemisphere. T(λ) is the photometric band of the device, that is, the normalized spectral transmittance of the whole setup including the spectral sensitivity of the irradiance-to-frequency converter, the spectral transmittance of the protective glass, filters, optical collector, and any other wavelength-dependent factor. T(λ) is a unitless function normalized to 1 at its maximum. The constant *K*, with units Hz/(Wm^−2^sr^−1^), provides the absolute link between the converter output frequency and the spectrally weighted and field-of-view averaged incident radiance. The dark frequency$f_{D}$ accounts for the output of the converter under complete darkness conditions.

According to Equation (1), the spectrally weighted and field-of-view averaged radiance L at the entrance plane of the detector,

(2) $$L=\int_{\lambda =0}^{\infty }T\left(\lambda \right)\;\left[\int_{\Omega }P\left(\omega \right)L_{\lambda }\left(\omega \right)\;d^{2}\omega \right]\;d\lambda$$

can be directly deduced from the converter output frequency, f, as

(3) $$L=G\left(f-f_{D}\right)$$

once the constant G≡1/K (Wm^−2^sr^−1^Hz^−1^) and the dark frequency $f_{D}$ (Hz) have been determined by calibration. A complete characterization of the radiometric properties of the detector also requires the precise measurement of the function T(λ) characterizing the device’s photometric band.

### 2.2. Radiometric Calibration

The radiometric calibration for determining G, T(λ), and $f_{D}$ can be performed by means of a spectrally tunable light source, an integrating sphere providing an angularly uniform radiance, and an auxiliary traceable calibrated photodiode. Note that, under angularly uniform radiance illumination of narrow spectral width Δλ around *λ*, Equation (2) simplifies to

(4) $$L=T\left(\lambda \right)L_{\lambda }\;\Delta \lambda \;\left[\int_{\Omega }P\left(\omega \right)d^{2}\omega \right]=T\left(\lambda \right)\;L_{\lambda }\;\Delta \lambda$$

(the normalization of P(ω) makes the integral equal to 1). The expected output frequency of the converter chip is then:

(5) $$f\left(\lambda \right)=KT\left(\lambda \right)L_{\lambda }\;\Delta \lambda +f_{D}$$

On the other hand, and under the same exposure conditions, the radiance $L_{\lambda }\;\Delta \lambda$ can be determined from the electric intensity $i_{\lambda }$ provided by the calibrated photodiode as

(6) $$L_{\lambda }\;\Delta \lambda =i_{\lambda }/\left(Q_{\lambda }\;S_{p}\;F_{p}\right)$$

where $Q_{\lambda }$ is the wavelength-dependent responsivity of the photodiode (units A/W), $S_{p}$ is the area of its illuminated surface, and $F_{p}$ is its effective field-of-view function (see Appendix A, for details). By substituting Equation (6) into Equation (5) we get

(7) $$KT\left(\lambda \right)=\left[f\left(\lambda \right)-f_{D}\right]\;Q_{\lambda }\;S_{p}\;F_{p}/i_{\lambda }$$

so that by measuring f(λ) and $i_{\lambda }$ across the relevant wavelength range, and once the photodiode responsivity, illuminated surface, and effective field-of-view function are known, the values of the product KT(λ) can be determined. Since T(λ), by definition, is normalized to 1 at its maximum, the value of the scaling constant is obtained as K=max[KT(λ)] and from this we obtain the value of G=K−1, and T(λ)=KT(λ)/max[KT(λ)]. The dark frequency, $f_{D}$, can be deduced from the intercept of the linear regression in Equation (7), or be directly measured under zero radiance conditions.

### 2.3. Formalizing the Absolute (AB) Astronomical Magnitudes Units System

For applications in astronomy and astrophysics, it is usual to express the radiance L (Equation (2)) in units of magnitudes per square arcsecond within the corresponding photometric band, in our case T(λ). The value of L in *T*-band mag/arcsec^2^, $m_{T}$, is defined as:

(8) $$m_{T}=-2.5\log_{10}\left(L/L_{r}\right)$$

where $L_{r}$ is a freely chosen, but explicitly stated, reference radiance that sets the origin of the *T*-band magnitude scale. From Equations (3) and (8) we can write:

(9) $$m_{T}=ZP-2.5\log_{10}\left[f(Hz)-f_{D}(Hz)\right]$$

where ZP, the ‘zero-point’ of the system, is given by:

(10) $$ZP=-2.5\log_{10}\left[G\left(Wm-{}^{2}sr^{-1}Hz^{-1}\right)\right]+2.5\log_{10}\left[L_{r}(Wm^{-2}sr^{-1})\right]$$

The value of ZP is of course contingent on the particular choice of $L_{r}$. This choice is arbitrary, as far as it is well-defined and is consistently applied. Different reference light sources have been traditionally used in astronomy to set the origin of the various magnitude (irradiance) scales used in this field [29]. Many of them are based on the spectral irradiance produced at the top of the Earth’s atmosphere by well-known stars like the Sun or Vega (α Lyr). A convenient system, not tied to any particular star, is the AB (absolute) magnitude scale [30,31], of which the reference source is defined as the one producing at the entrance plane of the detector a constant spectral irradiance, per unit frequency interval, equal to $E_{0}\left(\nu \right)=3631$ jansky (Jy) throughout the whole spectral domain (1 Jy = 10^−26^ Wm^−2^Hz^−2^). The associated reference source for the (radiance) scale of AB magnitudes per square arcsecond is the one producing this irradiance at the entrance plane of the measuring device under normal incidence per square arcsecond (i.e., $\Delta \omega_{0}$ = 1 arcsec^2^ = 2.3504 × 10^−11^ sr) of solid angle extent. Hence the spectral radiance of the reference source is $L_{0}\left(\nu \right)=E_{0}\left(\nu \right)/\Delta \omega_{0}$ (per unit frequency interval) or, equivalently, $L_{0}\left(\lambda \right)=\left(c/\lambda^{2}\right)E_{0}\left(\nu \right)/\Delta \omega_{0}$ (per unit wavelength interval), where c is the speed of light in vacuum. Note that, whereas $L_{0}\left(\nu \right)$ is constant, $L_{0}\left(\lambda \right)$ turns out to be wavelength-dependent due to the relationship $d\nu =\left(-c/\lambda^{2}\right)d\lambda$ between the frequency and wavelength differential intervals.

A uniform hemispheric light field with spectral radiance $L_{\lambda }\left(\omega \right)=L_{0}\left(\lambda \right)$ gives rise, according to Equation (2), to the spectrally weighted and field-of-view averaged AB reference radiance [32]

(11) $$L_{r,AB}=3631\;\left[\mathrm{Jy}\right]\;\times \frac{c}{\Delta \omega_{0}}\;\times \int_{\lambda =0}^{\infty }\frac{T\left(\lambda \right)}{\lambda^{2}}d\lambda$$

that will be used here to set the ${ZP}_{AB}$ zero point according to Equation (10). This zero point, in combination with Equation (9), defines the desired AB magnitude system corresponding to the detector’s T(λ) photometric band.

### 2.4. Experimental Calibration Setup

The radiometric calibration described in Section 2.2 was carried out in the Laboratory for Scientific Advanced Instrumentation (*Laboratorio de Instrumentación Científica Avanzada*, LICA) of Universidad Complutense de Madrid. The light source was a Quartz Tungsten Halogen from Oriel Corp. Narrow spectral intervals were selected using a CS260 monochromator from Newport Corp. with 1.3 nm resolution (FWHM). The light beams were fed into the entrance port of an Oriel barium sulfate integrating sphere with 20 cm diameter. This sphere has two output ports, fitted with identical Lambertian diffusers, to which the TESS-W or SQM-LU-DL sensors and the auxiliary traceable calibrated photodiode (Hamamatsu S2281) can be attached using specifically designed optomechanical mounts. These output ports provide a uniform angular radiance that completely fills the effective field-of-view of the TESS-W and SQM-LU-DL sensors. The working plane of the auxiliary photodiode, whose active surface has a diameter of 11.3 mm, is located at 9 mm distance from the corresponding diffuser. Since each output port has a diameter of 45 mm, the half angle subtended by the diffuser as seen from the center of the photodiode is *θ_max_* = 68.2° and the photodiode effective field-of-view function is $F_{p}=\pi \sin^{2}\left(\theta_{max}\right)=2.71$ sr.

The calibration measurements f(λ) vs. $i_{\lambda }$ (see Section 2.2 above) can be done either simultaneously, by locating the detector and the auxiliary photodiode in complementary ports, or sequentially, by using the same port for both. After several checks, and in order to avoid any potential bias derived from small asymmetries in the spectral optical outputs at each port, the final calibrations were performed sequentially. The light source was allowed to stabilize before starting the measurements of $i_{\lambda }$ from *λ* = 350 to 1000 nm in 10 nm intervals, the values of f(λ) were subsequently recorded for the same wavelengths, and the $i_{\lambda }$ measurements were repeated at the end of each session to ensure the source stability, found to be better than 0.25% averaged over the whole spectral interval (max 2.9%, min 0.0%). The measurements were performed at a lab temperature of 21 °C. Strict control was exerted to ensure that the readings were not affected by external stray light.

## 3. Results

### 3.1. TESS-W Detectors

**Figure 1:**
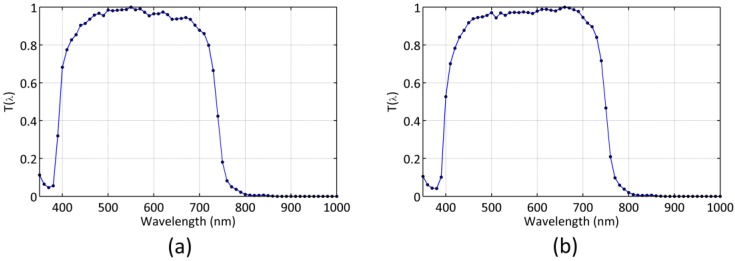
Normalized spectral sensitivity of the Telescope Encoder and Sky Sensor (TESS-W) night sky brightness meters (**a**) stars3, and (**b**) stars222

As an example of application, we performed the experimental calibration measurements and the calculations described above for two TESS-W units (stars3 and stars222). The resulting normalized spectral sensitivities T(λ) are displayed in Figure 1. The dark frequency $f_{D}$ recorded at laboratory temperature was equal to zero in both units for all practical purposes. The resulting calibration constants G, AB reference radiance $L_{r,AB}$, and zero point ${ZP}_{AB}$ corresponding to the TESS-W T(λ) photometric band, with their one-sigma uncertainties (*σ*), are listed in Table 1.

### 3.2. SQM Detectors

**Figure 2:**
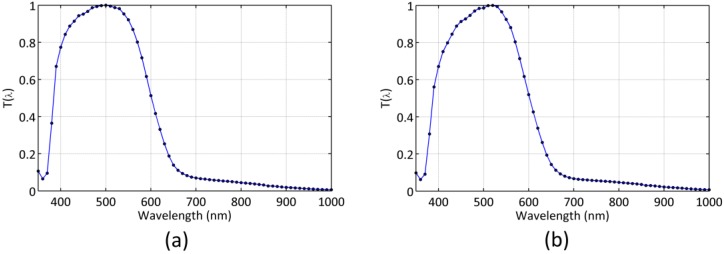
Normalized spectral sensitivity of the of the SQM USB enabled data-logging light meters (SQM-LU-DL) (**a**) #2370, and (**b**) #2747.

The same calibration procedure was applied to the readings of two SQM-LU-DL detectors, with serial numbers 2370 and 2747, respectively, whose resulting normalized spectral sensitivities T(λ) are displayed in Figure 2. Table 2 summarizes the values of their calibration constants and associated uncertainties (*σ*).

## 4. Discussion

The calibration results reported in Section 3 reveal both the similarities and the differences of the two types of widely used night sky brightness detectors. Any relative comparison of performance must take into account that the photometric bands T(λ) of the TESS-W and the SQM-LU-DL are different, and hence that they provide complementary information on the night sky radiance as measured in two overlapping but not coincident photometric channels. The calibration constant G (Wm^−2^sr^−1^Hz^−1^), that corresponds to the in-band detected radiance per Hz, is smaller for the TESS-W, what amounts to a higher sensitivity in absolute terms. The reproducibility of the calibration constants within each type of detector is fairly good, with constants G differing in the range 1–5%, and absolute AB zero points differing 0.03–0.04 mag_AB_/arcsec^2^. It must be noted, however, that these results are provided here as an example of application of the calibration procedure described in this paper, and that more extensive tests with larger samples should be performed before establishing the reproducibility of both families of instruments.

Regarding the experimental calibration measurements, it must be borne in mind that when the ambient temperature is high (>25 °C), the dark frequency $f_{D}$ may deviate significantly from zero [28]. The actual value of $f_{D}$ at the calibration temperature must be taken into account in the calculation of the calibration constants (G, $L_{r,AB}$, and, subsequently, ${ZP}_{AB}$) from the recorded lab measurements. Note however that these constants, once determined, are themselves independent from the operating temperature, which only appears implicitly in Equation (9) through the $f_{D}$ term. At typical nighttime low operating temperatures, the value of $f_{D}$ turns out to be in most cases negligible.

The absolute AB astronomical magnitude system is a well-established reference frame for the communication of scientific results. The TESS-W and SQM units actually operating in many places of the world routinely report their measurements in mag/arcsec^2^ using some variant of the Johnson V photometric system, with zero points ${ZP}_{m}$ provided by the manufacturer. These measurements can be directly converted to the AB system by adding to them a magnitude correction term $\Delta ={ZP}_{AB}-{ZP}_{m}$.

## 5. Conclusions

We present in this paper a general measurement model for the widely used night sky brightness meters based on irradiance-to-frequency semiconductor converters. We also describe their absolute calibration procedure, and the parameters that define the absolute radiance scale of AB magnitudes per square arcsecond, for their specific photometric bands, T(λ). As an example of implementation, we provide the calibration constants for several TESS-W and SQM-LU-DL detectors.

In order to establish well-defined and reproducible night sky brightness datasets, as well as to facilitate data sharing and inter comparison between different research teams, it is strongly suggested that the night sky brightness readings be expressed in band-weighted radiances (Wm^−2^sr^−1^) or, equivalently, in AB mag/arcsec^2^ with explicit reference to the precise definition of the specific photometric band of the measuring device.

## Figures and Tables

**Table 1:** Calibration constants of the TESS-W night sky brightness meters.

Constant	TESS-W Stars3	TESS-W Stars222	*σ*	Units
G	1.22·10^−6^	1.16·10^−6^	0.06·10^−6^	Wm^−2^sr^−1^Hz^−1^
$L_{r,AB}$	521.8	516.9	7.8	Wm^−2^sr^−1^
${ZP}_{AB}$	21.58	21.62	0.06	mag_AB_/arcsec^2^

**Table 2:** Calibration constants of the SQM-LU-DL night sky brightness meters.

Constant	Serial#2370	Serial#2747	*σ*	Units
G	1.51·10^−6^	1.49·10^−6^	0.08·10^−6^	Wm^−2^sr^−1^Hz^−1^
$L_{r,AB}$	433.9	415.4	7.9	Wm^−2^sr^−1^
${ZP}_{AB}$	21.15	21.12	0.06	mag_AB_/arcsec^2^

## Appendix A

In this appendix, some of the steps leading to Equations (1) and (6) are formalized. Irradiance-to-frequency photodiode converter chips like the TSL237 used in the TESS-W and SQM-LU-DL detectors provide an electric signal with output frequency f whose logarithm (once f is corrected from the dark frequency $f_{D}$) depends linearly on the logarithm of the spectrally weighted irradiance *E*′ incident on the chip, with unit slope and non-zero intercept [28]. This is equivalent to a linear relationship in natural units:

(A1) $$f-f_{D}=\int_{\lambda =0}^{\infty }R_{0}\left(\lambda \right){E_{\lambda }}^{\prime }\;d\lambda$$

where $R_{0}\left(\lambda \right)$ is the spectral responsivity of the converter, with units Hz/(Wm^−2^), and ${E_{\lambda }}^{\prime }$ is the spectral irradiance on its surface.

Let us denote by $x^{\prime }$ the two-dimensional position vector of a generic point on the chip active surface, and by $\omega^{\prime }=\left(\theta^{\prime },\phi^{\prime }\right)$ the two-dimensional vector describing a generic direction of incidence of the light rays in a spherical coordinate system centered at $x^{\prime }$ and with its *Z* axis normal to that surface. The spectral flux (Wm^−2^nm^−1^) incident on an infinitesimal patch $d^{2}x^{\prime }$ of the surface around $x^{\prime }$, due to a small bundle of rays of spectral radiance ${L_{\lambda }}^{\prime }\left(x^{\prime },\omega^{\prime }\right)$ and solid angle spread $d^{2}\omega^{\prime }$ incident along the direction $\omega^{\prime }$, is

(A2) $$d{\Phi_{\lambda }}^{\prime }\left(x^{\prime },\omega^{\prime }\right)={L_{\lambda }}^{\prime }\left(x^{\prime },\omega^{\prime }\right)\cos\theta^{\prime }d^{2}\omega^{\prime }d^{2}x^{\prime }$$

The overall spectral flux on the chip will be the integral of Equation (A2) spatially extended to its whole active surface S, and angularly extended to the Ω′ = 2π sr set of directions of its front facing hemisphere. The resulting spectral irradiance on S is then:

(A3) $${E_{\lambda }}^{\prime }=\frac{1}{S}\int_{S}\int_{\Omega^{\prime }}{L_{\lambda }}^{\prime }\left(x^{\prime },\omega^{\prime }\right)\cos\theta^{\prime }d^{2}\omega^{\prime }d^{2}x^{\prime }$$

Now let us recall that the light rays arrive to the chip after being deflected by the different elements of the optics of the TESS-W or SQM-LU-DL detector. The ray propagation can be characterized by a ray transfer Equation of the form $\left(x^{\prime },\omega^{\prime }\right)=\Gamma \left(x,\omega \right)$, which relates the position and direction of incidence (x,ω) of a ray at the entrance aperture of the detector to its values $\left(x^{\prime },\omega^{\prime }\right)$ at the plane of the chip. This Equation can be interpreted as a general coordinate transformation in the four-dimensional position-momentum ray space, with associated Jacobian determinant J(x,ω). On the other hand, the radiance at the chip, ${L_{\lambda }}^{\prime }\left(x^{\prime },\omega^{\prime }\right)$, is equal to the radiance $L_{\lambda }\left(x,\omega \right)$ of the corresponding rays at the entrance aperture of the detector, excepting for the attenuation due to the optics, so that we can write

(A4) $${L_{\lambda }}^{\prime }\left(x^{\prime },\omega^{\prime }\right)=\Lambda \left(x,\omega ,\lambda \right)L_{\lambda }\left(x,\omega \right)$$

where Λ(x,ω,λ) is the overall spectral attenuation function, that generally depends on the point of incidence and direction of propagation of each ray at the entrance aperture of the detector (determining its propagation path throughout the whole optical system), in addition to the wavelength. For sky brightness monitoring applications, it turns out that $L_{\lambda }\left(x,\omega \right)$, for each ω, is fairly constant across the surface of the detector entrance aperture, so that $L_{\lambda }\left(x,\omega \right)\equiv L_{\lambda }\left(\omega \right)$. Substituting this condition in (A4) and performing the change of coordinates in (A3), taking into account that $d^{2}\omega^{\prime }d^{2}x^{\prime }=J\left(x,\omega \right)d^{2}\omega \;d^{2}x$ and that $\cos\theta^{\prime }\equiv \cos\theta^{\prime }\left(x,\omega \right)$ we get:

(A5) $${E_{\lambda }}^{\prime }=\frac{1}{S}\int_{\tilde{S}}\int_{\Omega }\Lambda \left(x,\omega ,\lambda \right)L_{\lambda }\left(\omega \right)\cos\theta^{\prime }\left(x,\omega \right)\;J\left(x,\omega \right)\;d^{2}\omega \;d^{2}x$$

where $\tilde{S}$ and Ω denote the new limits of integration resulting from the change of variables.

In most cases of interest, the attenuation of the beam due to geometrical propagation factors will be independent of (or only weakly dependent on) the wavelength (e.g. in case of Fresnel reflections, only through the small wavelength dependence of the refractive index of the optical components), and the main spectral attenuation will be due to the bandpass filter and be approximately equal for all geometric rays. In this case the Λ function in Equation (A5) can be factorized as Λ(x,ω,λ)=Λa(x,ω)Λb(λ), and Equation (A5) can be rewritten as:

(A6) $${E_{\lambda }}^{\prime }=\Lambda b\left(\lambda \right)\;\int_{\Omega }L_{\lambda }\left(\omega \right)\;\left[\frac{1}{S}\int_{\tilde{S}}\Lambda_{a}\left(x,\omega \right)\cos\theta^{\prime }\left(x,\omega \right)\;J\left(x,\omega \right)\;d^{2}x\right]\;d^{2}\omega$$

We can now define the effective field-of-view function of the detector as:

(A7) $$P\left(\omega \right)=\frac{1}{P0}\left[\frac{1}{S}\int_{\tilde{S}}\Lambda_{a}\left(x,\omega \right)\cos\theta^{\prime }\left(x,\omega \right)\;J\left(x,\omega \right)\;d^{2}x\right]$$

where 1/P0 is the normalization constant required to fulfill the condition $\int_{\Omega }P\left(\omega \right)\;d^{2}\omega =1$. Hence, Equation (A6) becomes:

(A8) $${E_{\lambda }}^{\prime }=P_{0}\Lambda_{b}\left(\lambda \right)\;\int_{\Omega }P\left(\omega \right)L_{\lambda }\left(\omega \right)\;d^{2}\omega$$

whose substitution into Equation (A1) leads to:

(A9) $$f-f_{D}=\int_{\lambda =0}^{\infty }P_{0}R_{0}\left(\lambda \right)\Lambda_{b}\left(\lambda \right)\;\left[\int_{\Omega }P\left(\omega \right)L_{\lambda }\left(\omega \right)\;d^{2}\omega \right]\;d\lambda$$

and, by defining K and T(λ) such that $KT\left(\lambda \right)=P_{0}R_{0}\left(\lambda \right)\Lambda_{b}\left(\lambda \right)$ and max[T(λ)]=1 we finally get Equation (1) of the main text:

(A10) $$f=K\int_{\lambda =0}^{\infty }T\left(\lambda \right)\;\left[\int_{\Omega }P\left(\omega \right)L_{\lambda }\left(\omega \right)\;d^{2}\omega \right]\;d\lambda +fD$$

On the other hand, the electric intensity $i_{\lambda }$ at the output of the auxiliary traceable calibrated photodiode under quasi-monochromatic illumination with radiant spectral flux $\Phi_{\lambda }$ (W/nm) and bandwidth Δλ (nm), is given by

(A11) $$i_{\lambda }=Q_{\lambda }\Phi_{\lambda }\;\Delta \lambda$$

where $Q_{\lambda }$ is the wavelength-dependent photodiode’s responsivity (units A/W). The spectral flux over the photodiode, in turn, is the spatial and angular integral of the spectral radiance weighted by the zenith-angle cosine factor:

(A12) $$\Phi_{\lambda }=\int_{Sp}\int_{\Omega p}L_{\lambda }\left(x,\omega \right)\;\cos\theta \;d^{2}\omega \;d^{2}x$$

where $S_{p}$ is the area of the auxiliary photodiode active surface and $\Omega_{p}$ is the solid angle subtended by its field of view.

If the radiance incident on the auxiliary photodiode is spatially and angularly constant, i.e., if $L_{\lambda }\left(x,\omega \right)=L_{\lambda }$, Equation (A12) reduces to $\Phi_{\lambda }=S_{p}\;F_{p}\;L_{\lambda }$, with $F_{p}=\int_{\Omega_{p}}\cos\theta \;d^{2}\omega$. Substituting this last expression into Equation (A11) leads to:

(A13) $$i_{\lambda }=Q_{\lambda }\;S_{p}\;F_{p}\;L_{\lambda }\;\Delta \lambda$$

from whence Equation (6) immediately follows.
